# Optimization of T4 phage engineering via CRISPR/Cas9

**DOI:** 10.1038/s41598-020-75426-6

**Published:** 2020-10-26

**Authors:** Michelle M. Duong, Caitlin M. Carmody, Qinqin Ma, Joseph E. Peters, Sam R. Nugen

**Affiliations:** 1 Department of Food Science and Technology, Cornell University, Ithaca, NY 14853 USA; 2 College of Life Sciences, Sichuan Normal University, Chengdu, China; 3 Department of Microbiology, Cornell University, Ithaca, NY 14853 USA

**Keywords:** Bacteriophages, Microbial genetics, Phage biology

## Abstract

A major limitation hindering the widespread use of synthetic phages in medical and industrial settings is the lack of an efficient phage-engineering platform. Classical T4 phage engineering and several newly proposed methods are often inefficient and time consuming and consequently, only able to produce an inconsistent range of genomic editing rates between 0.03–3%. Here, we review and present new understandings of the CRISPR/Cas9 assisted genome engineering technique that significantly improves the genomic editing rate of T4 phages. Our results indicate that crRNAs selection is a major rate limiting factor in T4 phage engineering via CRISPR/Cas9. We were able to achieve an editing rate of > 99% for multiple genes that functionalizes the phages for further applications. We envision that this improved phage-engineering platform will accelerate the fields of individualized phage therapy, biocontrol, and rapid diagnostics.

## Introduction

Bacteriophages (phages) present a novel solution to several persistent problems in the fields of food safety, agriculture, and medicine. Phages are viruses that specifically infect bacterial species, resulting in lysis and self-propagation in a targeted and efficient manner. A panoply of previous studies suggest that phages have broad application toward a variety of pressing issues, including providing promising solutions in treating infectious diseases^1–6^, serving as the primary engine within biosensors to detect foodborne pathogens^7–13^, and acting as biocontrol methods within the agricultural and aquaculture supply chain^14–21^. For example, infectious diseases resulting from multidrug antibiotic resistant bacteria have been a particularly persistent issue that has been mitigated via phage therapy. More recently, genetically engineered phages were therapeutically used for the first time to treat a mycobacterial infection that displayed lower susceptibility to wild type phage treatment^22^. Altogether, there is strong supporting evidence suggesting that phages present promising alternative solutions to a diverse set of global problems.

The chief limiting factor in the widespread adoption of phages for medical and industrial purposes, however, is the arduous task of phage hunting^23^ and the limited host range of wild type phages. Being able to dispose of or dampen this limiting factor by efficiently bioengineering synthetic phages and effectively tailoring phage-bacteria target specificity will significantly broaden the impact of phage therapy in daily application. Early works prior to 2014 were successful in bioengineering a wide range of wild type phages, including K11, M13, PPO1, ɸX174, T3, and T7 phages, but at a very inefficient genomic editing rate of 5 × 10^–3^ (0.05%) or lower^24–27^. There has been notable progress in improving the genomic editing rate as the field steadily moves from traditional techniques for engineering phages such as classical homologous recombination to an improved recombineering approach with the introduction of *Lambda Red* (λ-Red) recombination^28,29^. Other innovative approaches include using type I-E CRISPR/Cas^30^ as a counter selection tool and more recently, whole phage genome reconstruction in vitro or within yeast^31,32^. While these techniques have certain useful applications, there are issues that commonly arise when they are used to engineer large DNA genomes due to the existence of modified bases, errors during the recombination of targeted regions, and the overall impurity of samples when isolating from in vitro systems. Despite representing novel approaches to bioengineering phages, these techniques remain inefficient, further highlighting the need for an improved method with higher editing efficiency.

One promising lead in this respect is CRISPR/Cas9 (clustered regularly interspaced short palindromic repeats-Cas associated protein 9), which has been a successful tool in engineering prokaryotic and eukaryotic systems. Given its success of genetic engineering in a variety of other models, the use of CRISPR/Cas9 has promising applications for phage engineering. The CRISPR/Cas9 system was successfully employed by the Moineau group to introduce point mutations, deletions, and insertions to a lactococcal phage p2^33^. More recently, CRISPR/Cas9 facilitated an insertion of a red fluorescent protein to a *Klebsiella* phage phiKpS2 with an efficiency of 87.5%^34^. However, there have been conflicting evidence regarding CRISPR/Cas9 effectiveness at editing phages with extensive base modifications. In 2017, Tao et al. also employed CRISPR/Cas9 to engineer T4 phages but found low and varying editing efficiency of 0.03–3%^35^. In addition, this group observed that CRISPR/Cas9 is ineffective at cleaving the cytosine hydroxylmethylated and glucosylated modified T4 genome; this contradicts an earlier finding by Church et al.^27^, highlighting a need to further evaluate the role of CRISPR/Cas9 in editing phages with modified bases.

**Figure 1:**
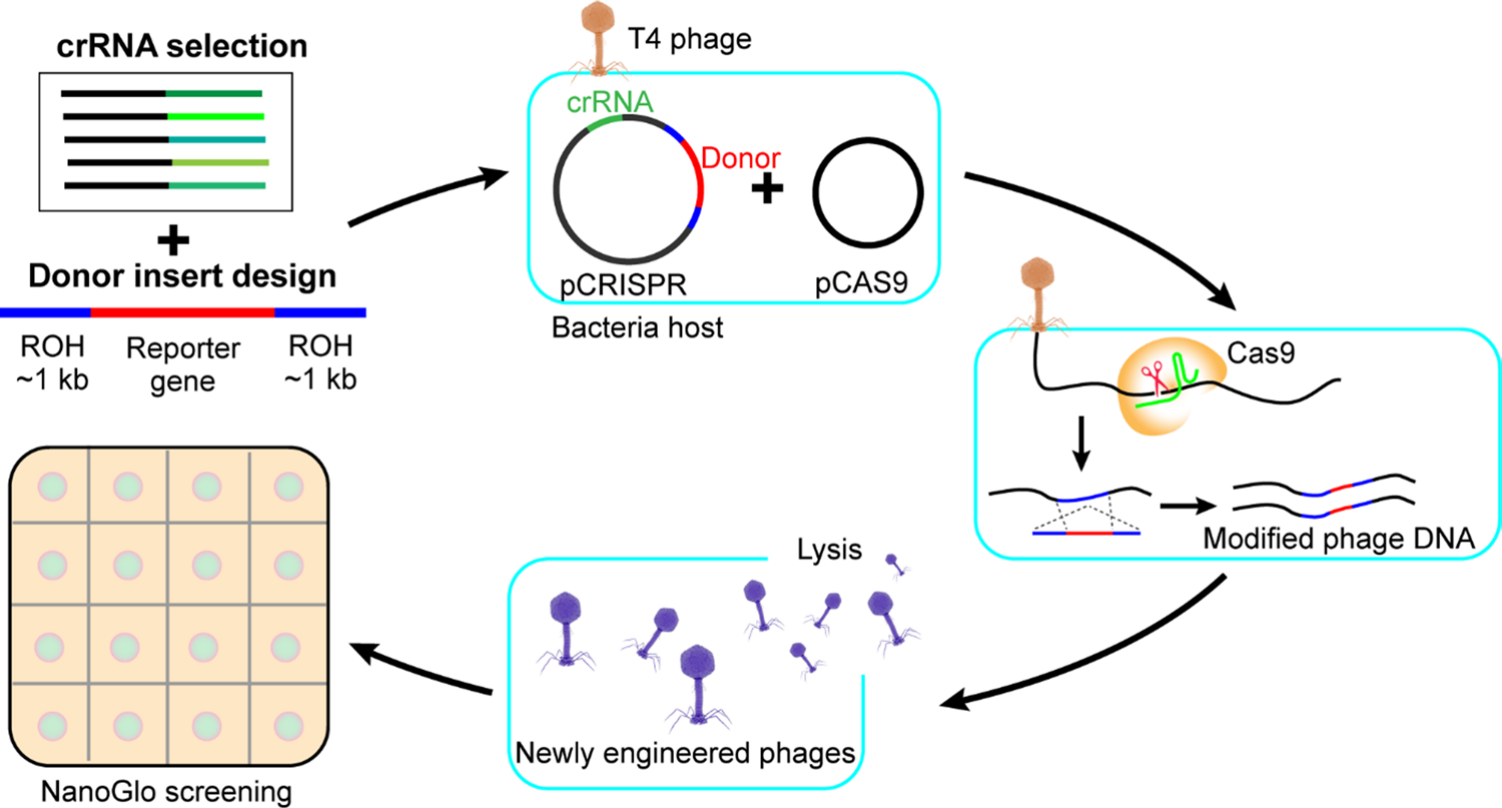
CRISPR/Cas9 T4 Phage Engineering Workflow. Candidate crRNAs targeting the T4 gene of interest were validated by a plaque assay. The most effective crRNA was selected based on largest reduction in Efficiency of Plating (EOP) and inserted into the synthetic CRISPR array in pCRISPR. The donor insert was designed to contain a reporter gene flanked by regions of homology to the crRNA recognition sequence and cloned into pCRISPR. A strain containing pCas9 and pCRISPR was infected with T4 phages. CRISPR/Cas9-mediated T4 genome cleavage followed by homologous recombination with the donor plasmid resulted in genomic incorporation of the reporter gene. NanoGlo screening was used for luminescent detection of recombinant phages by addition of the reporter enzyme substrate to phage plaques.

Here, we review the CRISPR/Cas9 phage engineering approach to broaden our understanding of this editing tool on T4 phages. We streamlined the CRISPR/Cas9 system in order to synthetically engineer T4 phages and provide a foundation for engineering in other phage families (Fig. 1). Our results indicate that the rate limiting factor in T4 phage engineering using CRISPR/Cas9 is crRNA selection; effective crRNAs can overcome barriers otherwise imposed by the DNA modifications in T4 phages. While exploring several CRISPR/Cas9 resources that were successful in engineering both eukaryotic and prokaryotic systems, we were able to achieve a recombination efficiency of > 99% in T4 phages.

## Results and discussion

### CRISPR/Cas9 plasmid selection contributes to cleaving success

To improve the efficiency of employing the CRISPR/Cas9 system to bioengineer T4 phages we reviewed and evaluated the pre-existing plasmids targeted for this system. Initially we genetically engineered T4 phages by using the single DS-SPCas (Addgene no. 48645) plasmid CRISPR/Cas9 system^35,36^ in which the crRNA sequences are individually cloned into this vector. This single plasmid system subsequently generates the Cas9:crRNA complex to cleave T4 phage DNA. Furthermore, we tested the duo plasmid system reported in Yaung et al. This group designed and employed the DS-SPCas (Addgene no. 48645) and the PM-SP!TB (Addgene no. 48650) plasmids in which Cas9 and the crRNA exist on separate plasmids^27,36^. Finally, we evaluated the dual plasmid system introduced by Marraffini et al. consisting of pCas9 (Addgene no.42876) and pCRISPR (Addgene no. 42875)^37^. In our early experiments we found that the single plasmid system and the duo plasmid proposed by Yaung et al. gave lower recombination frequencies, so we focused on the system designed by the Marraffini group which immediately gave higher rates of recombination.

### Assembly of a CRISPR RNA library via multiple scoring methods

An additional factor that we investigated in order to improve the CRISPR/Cas9 editing mechanism is selecting for potent crRNAs. Fundamentally, the Cas9 endonuclease within the CRISPR system cleaves DNA via a target sequence specific RNA guide. Cas9 cleavage activity is dependent on the crRNA sequence base paring with the target DNA^38,39^, hence developing a comprehensive crRNA library and selecting for high cleaving efficiency will improve the downstream probability of homology directed repair to generate mutant phages. Here we pooled findings from both eukaryote and prokaryote crRNA modeling systems to maximize the on-target binding (high sensitivity) and minimize the off-target binding (high specificity) of the crRNA to recognize and target the cut site^40–42^. The design of crRNA contained three specific considerations: 1) Protospacer adjacent motif (PAM) site, which allows us to identify all possible Cas9 cleavage sites within our region of interest; 2) Zhang specificity score to evaluate off-target binding; and 3) Doench activity score for on-target binding. Geneious (Biomatters, Ltd., Auckland, NZ), was used to streamline crRNA comparison and selection. Our three preconditions allowed us to develop a comprehensive library of crRNA candidates. The majority of crRNAs from our library have an 100% specificity score but vary in their Doench score on a 0–1 scale. Therefore, we selected the crRNAs with the highest Doench score in an attempt to maximize crRNA recognition and targeting efficiency (Table S1).

### Scoring methods are not good predictive measurements for selecting effective crRNAs

**Figure 2:**
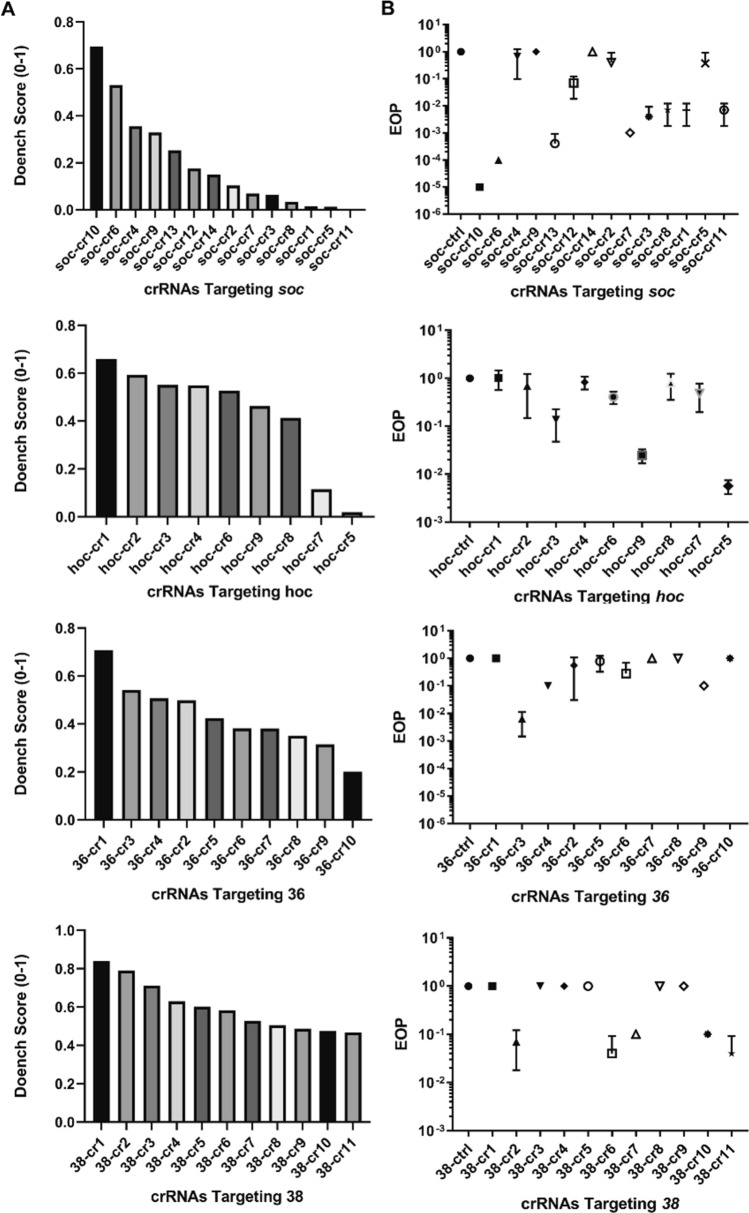
Doench scoring is not a good predictive measurement for selecting effective crRNAs. Experimental efficacy of DNA cleavage via EOP as evaluated in the *soc, hoc, gp36,* and *gp38* genes in T4 phages does not align with theoretical projection. (**A**) Doench scoring (0–1) to theoretically assess crRNAs on-target activity and off-target sites in which values closer to 1 resemble the most potent crRNA. The crRNAs are ranked in descending efficacy order. (**B**) Validation of the crRNAs’ theoretical selection via plaque assay as demonstrated by the EOPs for *soc*, *hoc*, *gp36,* and *gp38*. Error bars indicate standard deviation of three experimental replicates. Lower EOP value represents better T4 phage DNA cleavage.

We screened 44 crRNAs spanning across four different genes in T4 phages, including both essential and non-essential genes. Due to their utility in synthetic phage therapy, phage display, and host range expansion, the *soc* (small outer capsid), *hoc* (highly immunogenic outer capsid), *gp36* (long tail fiber gene 36), *gp38* (tail fiber adhesin gene 38) were selected for this study. Among the three theoretical preconditions for selecting crRNAs, initial experiments suggested the Doench score was the only precondition that provided a comparative analysis tool in determining crRNA strength. All 44 crRNAs used have the highest Doench score among the ~ 200 possible crRNA sites identified across four tested genes, dovetailing with our original hypothesis that computational scoring through use of the Doench score is a good predictive indicator. However, through our experiments we discovered that the Doench score did not serve as a good computational predictor of crRNA targeting efficiency for T4 (Fig. 2). For instance, as illustrated with *hoc,* there was zero log drop in EOP among six of the nine tested crRNAs despite the Doench score for these crRNAs predicting a high cutting efficiency. Interestingly, the crRNA with the lowest Doench score had the highest cutting efficiency resulting in a 3 log drop in EOP. This lack of correlation between Doench score and cutting efficiency is consistently observed among the four genes that we evaluated. As a result, we found no correlation between crRNAs efficacy as determined by plaque assays relative to that of the Doench score.

The Doench score was developed in murine and human model systems and it is possible that the Doench score’s predictive capability cannot be extended to phages. We recognize that there are other non-phage crRNA predictive models that we did not evaluate and their application to phage genomes remains unknown. Concurrently, we did not identify any distinguishable characteristics in the nucleotide composition between the efficient and inefficient crRNAs that were screened. Factors such as crRNA accessibility to the targeted area due to DNA modifications, formation of crRNA secondary structures, phage specific on-target and off-target crRNA scoring, or viral genome variations are all constructive elements that will help to design a better predictive model for phage genome editing via CRISPR/Cas9.

This notwithstanding, an alternative modeling system that is effective for selecting crRNA directed at phage DNA does not currently exist. We were able to nonetheless experimentally screen for efficient crRNAs as measured in a log reduction of EOP. Among the four genes, we were able to identify a potent crRNA for *soc*, in which it induced a 5-log reduction in EOP, an indicator that the crRNA is effective at targeting and directing Cas9 to induce a DNA double-strand break (DSB) hence killing T4 phages resulting in fewer plaques. crRNA screening from *hoc, gp36,* and *gp38* were less impressive with observable 1–3 log reduction in EOP (Fig. 2). Furthermore, we chose the best crRNA among the ones screened for *soc* and *hoc* to further evaluate how well CRISPR/Cas9 can assist homologous recombination to generate mutant T4 phages as indicated by the recombination frequency. Since a majority of the crRNAs screened presented a 1 log drop in EOP, we further investigated how efficiently the CRISPR/Cas9 assisted genome edit would perform with a weak crRNA; we evaluated this using *hoc* crRNA.

We suggest a shotgun approach to screen and select crRNAs for future workflows: a minimum initial screening of five crRNAs for each target site would in most cases circumvent the selection of an ineffective crRNA. This shotgun approach is preferred since 55% of the crRNAs tested in our study exhibit zero cutting activity, so there is a need to widely screen in order to select active crRNAs. A potent crRNA such as the one we have identified for *soc* will not only help with improving recombination but also serves as a strong negative selection tool against wild type phages post lysis.

### CRISPR/Cas9 assisted homologous recombination and negative selection resulted in > 99% efficiency at editing *soc* and *hoc*

**Figure 3:**
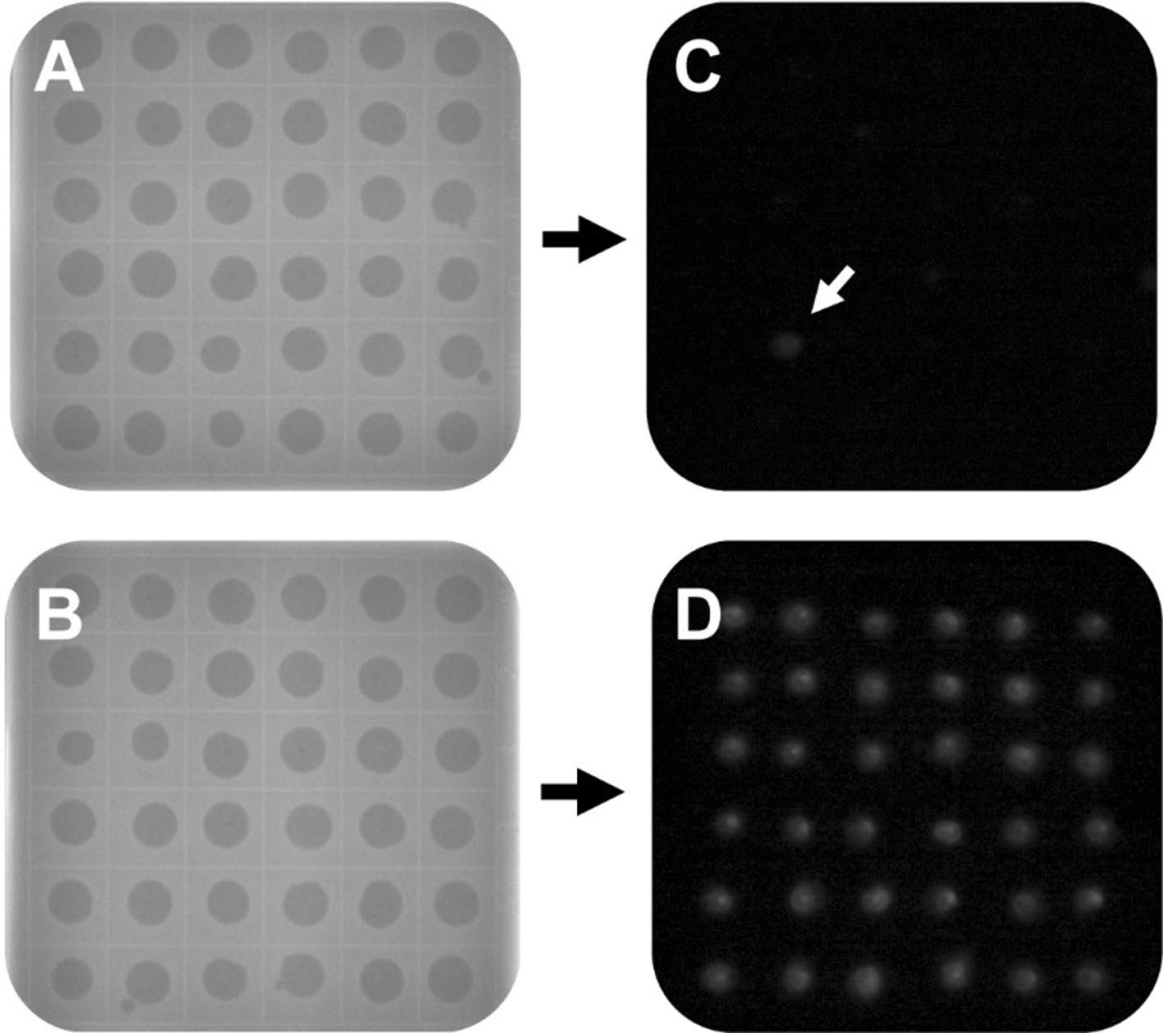
*nluc* insertion in T4 phages provides visual screening for recombinant phages. Comparing the efficiency of homologous recombination versus CRISPR/Cas9 assisted recombination. The schematic outlines the double plaque assay of T4 phage infection (**A**) of homologous recombination and (**B**) of CRISPR/Cas9 assisted recombination (representative images shown). The Nano-Glo luciferase assay system on the right demonstrates successful engineering of T4 phages (**C**) for homologous recombination (arrow) and (**D**) for CRISPR/Cas9-assisted recombination.

Effective selection of crRNA for the gene of interest is a major rate limiting step in facilitating and improving the downstream rate of homologous recombination (HR) and present as an efficient negative selection tool. Hence, an increase in DNA double strand breaks (DSBs) (as measured via EOP) results in an increased probability of homology-directed repair (HDR) in the presence of a donor template, allowing modifications to occur. CRISPR/Cas9 gene editing technology can be targeted to create a specific DSB within a gene of interest. We assessed the ability of CRISPR/Cas9 targeted cleavage in T4 phages to facilitate homology directed repair resulting in reporter gene insertion. We identified and selected the most effective crRNAs to cut *soc* or *hoc*. In addition, we designed ~ 1000 base pairs of homologous arms on either side of the inserted reporter gene *nanoLuc luciferase* (*nluc*) to improve editing efficiency^35^. This gene was selected for its luminescent capabilities to permit phenotypic observation of recombinant phages. *nluc* flanked by regions of homology to *soc* or* hoc* was cloned into the pCRISPR plasmid containing the crRNA targeting *soc* or *hoc*. We also introduced synonymous mutations to the crRNA sites on the pCRISPR plasmid to prevent Cas9-mediated cutting within the donor region of all knock-in studies; a negative selection tool to efficiently vet for mutant phage candidates. The pCRISPR (crRNA + donor) plasmid was co-transformed with pCas9 into* E. coli* DH5α to equip cells with the CRISPR/Cas9 gene editing machinery. These cells were then infected with wild type T4 phages. Of the resulting plaques, approximately 500 were randomly selected and screened for recombinants using a luciferase assay (Fig. 3). To account for natural homologous recombination, *E. coli* DH5α containing only pCRISPR (crRNA + donor) was used as a control.

**Figure 4:**
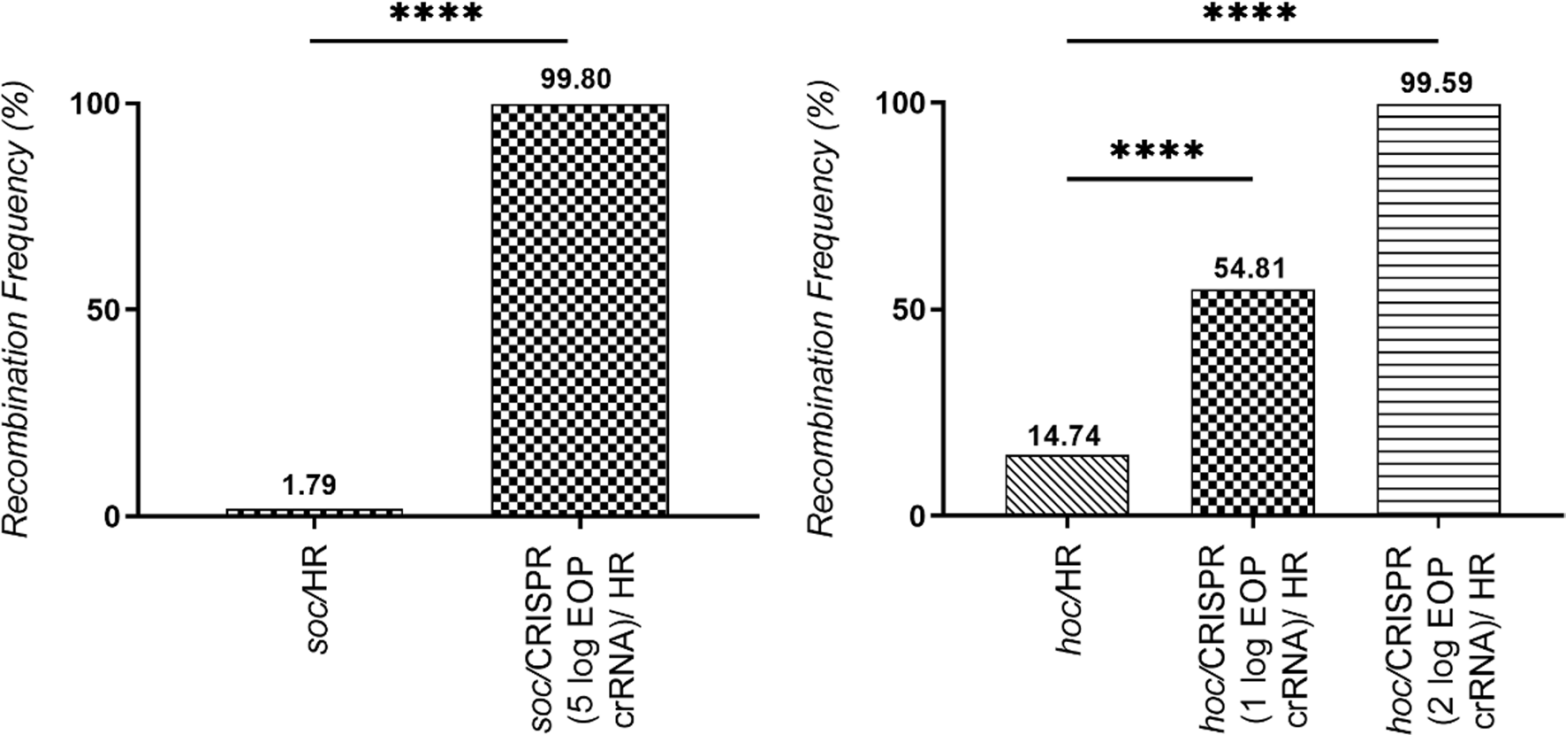
CRISPR/Cas9-assisted recombination resulted in > 99% recombination frequency. (**A**) *soc* was engineered using the CRISPR/Cas9 assisted platform with a 5 log EOP reduction crRNA, resulting in the production of > 99% recombinant phages. The CRISPR/Cas9 assisted recombination frequency is significantly higher than the natural homologous observed in the control (*p* < 0.0001, n = 1007 plaques) (**B**) *hoc* engineered using the CRISPR/Cas9-assisted platform with a 1 log EOP reduction crRNA and a 2 log EOP reduction crRNA also resulted in significantly higher recombination frequency than the control (*p* < 0.0001, n = 1102 plaques). Asterisks indicate significance (**** = *p* < 0.0001) by Chi-square Test.

The dual role of CRISPR/Cas9 in the experiments here is first, to induce a double-strand DNA cleavage that facilitates *nluc* insertion, and second, to act as an efficient negative selection tool, both roles resulting in 99.80% and 99.59% recombinants from the respective* soc* and *hoc* deletion. The natural homologous recombination control produced a higher than expected number of recombinants, 1.79% and 14.74% for *soc* and *hoc* respectively. This could be due to T4 phage's recombination-dependent DNA replication pathways such as the “join-copy” and “join-cut-copy” pathways used for starting replication forks during the infection process^43,44^. Even with high amounts of natural homologous recombination, CRISPR/Cas9-assisted homologous recombination resulted in significantly higher homologous recombination rates than the control (*p* < 0.0001) for both* soc* and *hoc*, demonstrating that CRISPR/Cas9 can be an useful tool in T4 phage genetic engineering with proper crRNA selection (Fig. 4). Ultimately, screening for recombinants without Cas9-directed cleavage showed significantly lower recombinants following screening of > 1000 isolates; however, using our vetted crRNA candidates, > 99% of the isolates were correct. Overall, we successfully created four mutant T4 phages using the CRISPR/Cas9 assisted genome engineering system labeled NRGp16, NRGp17, NRGp18, and NRGp20. The NRGp16 and NRGp17 phages each have a *soc* deletion with a replacement by either *nluc* or *nluc:CBM* (Nluc with a C-terminus carbohydrate binding module fusion) respectively. The NRGp18 phages have a *hoc* deletion with a *nluc:CBM* replacement. The NRGp20 phages have an N-terminus CBM fusion to the Hoc protein. Nluc:CBM has previously been shown to be a sensitive reporter for detecting *E. coli*^45^ and therefore is a pragmatic insertion to functionalize the phages for downstream applications. Genetic engineering of all four phages were confirmed through whole genome sequencing. The mutant T4 phages developed in this experiment were able to infect targeted hosts and propagate to generate similar end titers as the wild type.

## Conclusion

We have streamlined the CRISPR/Cas9-mediated genome engineering method and utilized it to efficiently edit T4 phages. More specifically, we developed an effective crRNA library against both non-essential and essential genes in T4 phages (*soc*, *hoc*, *gp36*, and *gp38)*. Furthermore, the crRNAs were successfully used within the CRISPR/Cas9 system to facilitate homologous recombination and generation of mutant T4 phages at an impressive rate of > 99%, well above that of past methods, which only achieved a rate of 0.04–0.3%. Although the Doench scoring system, one of the predictive methods we explored, was shown to be a nonideal benchmark for selecting effective crRNAs, we were still able to achieve approximately 50% recombination frequency with ineffective crRNAs that have a demonstrated EOP of 1 log reduction. We successfully translated this technique to create T2, T6, and P22 mutant phages (data not shown).

The recombination efficiency rate we achieved through the use of CRISPR/Cas9-mediated system has broad application for phage genome engineering, including experiments broadening host range, further functionalizing phages for use in biosensors, optimizing individualized phage therapy and many more applications. In addition, our method incorporates an efficient CRISPR-based counterselection system to produce a recombination efficiency rate consistently > 99%. Multi-loci edits are also possible, reducing the need to perform step wise phage genome engineering. As with other in vivo methods, CRISPR/Cas9-assisted phage engineering does not circumvent the host toxicity issue. This is especially relevant when the engineered region encodes a toxic gene that is harmful to the bacteria host, a factor that complicates phage genome engineering.

While the use of phages to combat potentially harmful bacteria dates back decades, the use of genetically engineered phages is still in its infancy. The combination of effective and efficient engineering of wild type phages granted by CRISPR/Cas9 removes a chief current limitation in generating synthetic phages for broader applications. The ability to genetically engineer phages will not only provide new and novel tools in this fight, but also advance our general understanding of phages and their host interactions.

## Materials and methods

### Cultures, materials, and reagents

*E. coli* DH5α and T4 phages were obtained from ATCC (Manassas, VA USA). Both plasmids pCAS9 (Addgene no.42876) and pCRISPR (Addgene no. 42875) were obtained from Addgene (Watertown, MA, USA). Bacteria overnight cultures (37 °C, 150 rpm, 17 h) were grown in Luria–Bertani (LB) broth with the appropriate antibiotic (50 µg/mL Kanamycin for pCRISPR, 25 μg/mL Chloramphenicol for pCas9). Both plasmids pCAS9 (Addgene no.42876) and pCRISPR (Addgene no. 42875) were obtained from Addgene (Watertown, MA, USA). All ligation enzymes and reagents were purchased from New England Biolabs (Ipswich, MA USA) unless otherwise indicated. All electro-competent cells were made in house according to standard lab protocol. T4 phages were propagated and maintained as described by Bonilla et al.^46^ Nano-Glo luminescent reagent was purchased from Promega (Madison, WI, USA) and prepared immediately before use according to the manufacturer’s recommendations. Luminescent images from the Nano-Glo assay were captured using a DSLR camera (Rebel T6, Canon, Melville, NY, USA) set to 30 s exposure in a dark box (LTE-13, Newport Corporation, Irvine, CA, USA).

### crRNAs design and CRISPR/Cas9 targeting plasmids

The crRNAs were designed and compiled via Geneious (Biomatters, Ltd., Auckland, NZ). The resulting crRNAs were filtered based on two criteria: 1. Doench score (0–1, highest values closer to 1 were chosen); 2. Zhang Specificity Score (0–100, all crRNAs chosen were 100).

CRISPR/Cas9 targeting plasmids consist of 1. pCAS9 (a Cas9 nuclease expression plasmid), and 2. pCRISPR (a crRNA expression plasmid for targeting a specific sequence). crRNAs were individually cloned into pCRISPR based on the method described by Marraffini et al.^37^. Each ligated product was transformed into house made electrocompetent DH5α cells. Correctly assembled pCRISPR with respective crRNA were screened and confirmed via colony PCR and Sanger sequencing. All crRNAs used in the experiment are listed in Table S1 of the supplemental material.

### Donor plasmid construction

Donor DNA expression cassettes containing *nanoLuc luciferase* (*nluc*)*, carbohydrate binding module* (*CBM*), and regions of homology to *soc* and *hoc* in T4 phages were codon optimized for *E. coli* and synthesized as gBlocks (IDT, Leuven, Belgium). Stitching regions were added via PCR to the respective gBlocks to ensure successful Gibson Assembly Cloning and annealed to pCRISPR via NEBuilder HiFi DNA Assembly per manufacturer’s instructions. All constructed donor plasmids were screened and confirmed via colony PCR and Sanger sequencing. For nucleotide sequences information, see Fig. S1. Refer to Table S2 for all primers used in NEBuilder HiFi DNA Assembly.

### Efficiency of plating

The efficacy of crRNA to cleave the gene of interest was determined by measuring the reduction in efficiency of plating (EOP). An overnight culture (200 µL) of *E. coli* DH5α containing pCRISPR (crRNA) and pCas9 or control pCRISPR (no crRNA) and pCas9 were added to molten 0.8% LB top agar containing the appropriate antibiotics and mixed. Then T4 phage dilutions (100 µL) previously determined by drop assay to achieve single plaques for each condition were added to the same molten tube, mixed, and poured onto an LB plate. Plates were incubated overnight at 37 °C. The EOP was calculated by dividing plaque forming units (PFU) produced by the input PFU. The EOP for each crRNA was compared to the control to determine the reduction in EOP.

### Homologous recombination rate screening

#### Day 1: creating T4 mutants

Overnight culture (200 µL) of *E. coli* DH5α containing pCRISPR (gRNA + donor) and pCas9 or only pCRISPR (gRNA + donor) were added to molten 0.8% LB top agar containing the appropriate antibiotics and mixed. 100 µL of 10^6^ PFU/mL WT T4 phages were added to the same molten tube, mixed, and poured onto an LB plate. Plates were then incubated overnight at 37 °C.

#### Day 2: background removal

A spot over assay was done via plaque transfer to minimize endogenous background from the plasmid containing DH5α. Overnight culture (200 µL) of *E. coli* DH5α without any plasmids was added to molten 0.8% LB top agar, mixed, and poured onto a gridline square LB plate. A sterile 10 µL pipette tip was used to transfer plaques resulting from the previous day's plaque assay over to the gridline square spot assay plate. Plates were then incubated overnight at 37 °C.

#### Day 3: screening for T4 mutants

Nano-Glo reagent (2 µL) was added directly onto each plaque on the gridline square spot assay plates. Plates were imaged in a dark box using a DSLR camera set to 30 s exposure to capture luminescence. Plate images were analyzed with ImageJ particle analysis software to count luminescent plaques.

### ImageJ analysis

ImageJ software was used to analyze luminescent plate photos. All images are converted to 8-bit with the threshold set to 10–20%. To identify plaques as positive or negative for luminescence, the particle analyzer feature was used with a size range of > 400 and no cut off for particle shape irregularity. DH5α cells were used as controls to identify threshold values for background luminescence. Refer to Table S3 for raw ImageJ data.

## Supplementary information

Supplementary Information.
