# Supplementary material for: Optimization of T4 phage engineering via CRISPR/Cas9
Source: Sci Rep. 2020 Oct 26;10:18229. doi: 10.1038/s41598-020-75426-6 (PMC7588440; doi:10.1038/s41598-020-75426-6)
Supplement: Supplementary file 1 — Supplementary Information. [file 41598_2020_75426_MOESM1_ESM.docx]

**Optimization of T4 Phage Engineering via CRISPR/Cas9**

Michelle M. Duong^1^, Caitlin Carmody^1^, Qinqin Ma^1,2^, Joseph E. Peters^3^, Sam R. Nugen^1*^

^1^Department of Food Science and Technology, Cornell University, Ithaca, NY, 14853, USA

^2^College of Life Sciences, Sichuan Normal University, Chengdu, China

^3^Department of Microbiology, Cornell University, Ithaca, NY 14853, USA

*Corresponding author: snugen@cornell.edu

**Supplementary Material**

Table S1. crRNA sequences organized by selected gene of interest

| Gene | crRNA | Sequence 5'-3' |
| --- | --- | --- |
| *soc* | soc-cr1 | AGTAACTGGTTAATAACTCA |
|  | soc-cr2 | GAAGATTTGTGGAAAGTAAC |
|  | soc-cr3 | TTGCTGCAAATGAAGATTTG |
|  | soc-cr4 | AAGAAAATCAACGTACTGAA |
|  | soc-cr5 | GAATATGCTGCTTTTTCTGA |
|  | soc-cr6 | AATATGCTGCTTTTTCTGAA |
|  | soc-cr7 | CTTTTTCTGAAGGGAATGTC |
|  | soc-cr8 | TCTGACGTTCACAAAATTTC |
|  | soc-cr9 | TTGTGAACGTCAGAATAAAG |
|  | soc-cr10 | TGTGAACGTCAGAATAAAGA |
|  | soc-cr11 | AAATAAGAAAATTGAAGGAA |
|  | soc-cr12 | GATGGAAATAAGAAAATTGA |
|  | soc-cr13 | ACATTTGAGCAGAAATTAGA |
|  | soc-cr14 | AATTACATGGCTAGTACTCG |
|  |  |  |
| *hoc* | hoc-cr1 | GCAGGTCCTTTTAGTACATA |
|  | hoc-cr2 | CGTAACTCCTGCTAGTCCTG |
|  | hoc-cr3 | TTAATAGTCTTTTGACCGGC |
|  | hoc-cr4 | GGCAGCAGTAAATTGAACTG |
|  | hoc-cr5 | TGTATCCATTACGCGATTCT |
|  | hoc-cr6 | ACCAGATGTAAGCTGAGCTA |
|  | hoc-cr7 | ACGTTAGTTGACCCTTCTAC |
|  | hoc-cr8 | ACACCCAGTGGTCAAACTGG |
|  | hoc-cr9 | GTTTCATCAATAACCCCTGT |
|  |  |  |
| *gp36* | 36-cr1 | AATTTTCCATTGAATCCAGC |
|  | 36-cr2 | AAAGAAAAAGGGAACCCGAA |
|  | 36-cr3 | ATAGTATTTATAAAGAAAAA |
|  | 36-cr4 | ACAATGGCTGATTTAAAAGT |
|  | 36-cr5 | TAGAGTACATCGTCACCGGC |
|  | 36-cr6 | TAAATACTATTCAAATAAAG |
|  | 36-cr7 | TCAAATAAAGGGGCATACAA |
|  | 36-cr8 | ATAAATACTATTCAAATAAA |
|  | 36-cr9 | AAAGTAGGTTCAACAACTGG |
|  | 36-cr10 | TTAAAAGTAGGTTCAACAAC |
|  |  |  |
| *gp38* | 38-cr1 | AGAACCTAAAGAAGGATACG |
|  | 38-cr2 | GTATAAATATCTTAAAAGGA |
|  | 38-cr3 | TTGAATGAGCAGGAAGACCG |
|  | 38-cr4 | ATATATGGACTGATGACGGT |
|  | 38-cr5 | TCTATTATAAAAACCTTAAG |
|  | 38-cr6 | GTCCATATATCAAATTCGCC |
|  | 38-cr7 | ATTTATGAAGAAGACCATCG |
|  | 38-cr8 | GGAAGACCGAGGCCTTTAAC |
|  | 38-cr9 | GAAAAGCATGAGCATTTAGG |
|  | 38-cr10 | TTTGATATATGGACTGATGA |
|  | 38-cr11 | ATATATTTTCATATTTAGAA |

Table S2. Primers used in NEBuilder HiFi DNA Assembly to Construct Donor Plasmids

| **Primer Names** | **Sequence** | **Description** |
| --- | --- | --- |
| NRG-975 | GAGTTTTCGTTCCACTGAGCGATGTTGTTTCTTGGATGC | soc-Donor-F-Hifi |
| NRG-976 | GATCTTTTCTACGGGGTCTGCGCACTTTCGTCCGTAAT | soc-Donor-R-Hifi |
| NRG-979 | GCTCAGTGGAACGAAAACTC | pCRISPR-R-Hifi |
| NRG-980 | CAGACCCCGTAGAAAAGATC | pCRISPR-F-Hifi |
| NRG-1310 | ACGTGAGTTTTCAACTAATTCCTTTTTAATCAAAGAAGG  ATTATC | hoc-Donor-F-Hifi |
| NRG-1315 | GCTCAGTGGAACCCAGTTGCCATATCTGAAG | hoc-Donor-R-Hifi |

Figure S1. Nucleotide Sequences for Donor Constructs

**1. NRGp16 insertion sequence *(nluc* T4Δ*soc)* 2553 bp**

**ROH up hoc—nluc—ROH down soc**

GATGTTGTTTCTTGGATGCGTGAACAAAAAGACTGTATTGATGATGAATTCCGCGAACTTCTGACTTCTCTTGGTGAAATGTCACGTGGTGAAAAAGAAGCTTCTGCTGTATGGAAAAAATGGAAAGCACGTTATATTGAAGCGCAAGAAAAACGCATTGATGAAATGTCCCCCGAAGACCAGCTCGAAATTAAATTTGAGCTTGTGGATATATTTCATTTCGTATTAAATATGTTTGTTGGCCTTGGAATGAATGCGGAAGAAATCTTTAAACTTTATTATCTGAAGAACAAACATAATTTTGAACGTCAAGATAATGGATATTAAACAAAAATTTTATAGAACATATATAGTAAAAGTTAGGACGCCGAAAGGCGTCTTTTGGTACGCTGGGAAACATGAATCATTTATTGTAAATCCATATAATGATAAATATCCGGGTTCAGGTAAAATACTATGGAACATATATCGTAAGTATGGATTTAATTATAAAATACGATGGTCAAAATGCCATGGTTCTAGAGAAAAATCATATGAAGTTGAACGTGAGCTAATATCTGCATTAAAACGTAAACACCCAGATACTTGCATTAATATTTCTCCTGGTGGTCAGGGTGGAGAAGGAAGAAAATGGACTGAGCAACAACGATTAGAACATAAACTTAGATTAAACAATCCTGAAACAAAAACTCGGATGAAGAATTCACAACGTATAGCCCAAAATAGGGCAGAAAGAAAAGCTCGGCAATCTGAAGTAATGAAAAAGTTTTATTCGAATGGCGGAAATAAAAAGATTTCAGAAGGAACTTCAAGGGCGCAAAGAAAAGCACCGCATTGGCATGAACCACTTAAAAGCGAAATACACGAGTTATGGGTTTCTTTAGGTAAACCAGCAACAGGCCCGGTTGTAAAGGCGCTTAAAGGAAAATATGATGTAACAAGTTCGGCTCTTAAGAATTTAATTTACTTATTCAGAAAAGAAGATGTATAAATAATCATGTAATTTAAATAAAGGAGAATTACATGGTATTCACTTTGGAGGATTTCGTAGGTGACTGGCGCCAGACAGCCGGTTACAATTTAGACCAAGTACTGGAACAGGGAGGTGTCAGTTCACTTTTTCAAAATTTGGGCGTTTCGGTTACACCCATCCAGCGCATTGTCCTTAGTGGAGAAAACGGGCTTAAAATCGACATCCATGTTATTATCCCCTATGAGGGGCTGTCGGGGGATCAGATGGGTCAGATTGAGAAAATCTTCAAAGTCGTCTACCCGGTTGATGACCATCACTTCAAGGTTATTCTTCATTACGGCACGCTGGTCATTGATGGCGTTACGCCTAATATGATTGACTACTTTGGCCGCCCCTATGAAGGGATCGCAGTGTTCGACGGCAAGAAGATCACTGTAACTGGAACATTGTGGAATGGCAATAAAATCATTGACGAACGTCTGATTAACCCGGACGGCTCTTTGCTGTTTCGTGTTACGATCAACGGGGTCACGGGCTGGCGTCTTTGTGAGCGCATTTTGGCTTAATAACTCAAGGACTCCTTCGGGAGTCCTTTTTTCATTTAAATGGTTTACTTTCCAAAATGAGTATGGTATAATAGAATTATCTTATAGAGGAGAGTACTATGTTAAATCGTTGGATTAAACCAAATGAAGATTTAGATATTATCATTTCACGACATGTAATGAAGAAATATGAACTACAACCATGGTCTACAGAAGTTGTTGTGCATTCATTTATGATGTACGCAGATGGTTCTGTCGAATTTAATGTAGAGATTCGATATGATTATGGTGAGAAGCAAGTCGAATTCAAAAGAGGCTTTTTGTAATGTTTATCTTTAATTGGTTTAAAAGTTTCTTTACGGATTTTTTCTCTACAACTCCTGGGGAAGGTGTAGTTCCTATTTCAAATGACTACCTTCCTTTAACTGTAGTTGAATATGTTTATATGGGAGATGGAACAGTAGAAGCAGTTACTATGACTTATGAAGAAGCCCAAGAATATTATAAAAATCCTTGGCGCTGGTCAACACCTACTACATCATCTAACACACAGAATACACAGTCTAGTTCTGATTCATATGATACTAATGTTCCTGTTCATGTATGGGCAGGTGATTCATGTGGAAGTTCTTGTGATTCTAGTTGTTCATCTACATCTTGTGATTGAGGAAAATTATGGAAGCAATTTTGTTTGAAATGTCTATCAATAGTATTAGTATGGCGTTTGTCAAGGATGTTCCAATTACTGTAGCAGTAATGATTGATAAAAGATATGACAATAATATGTATCTAGTAGAAGATTTTATTTCAATGCCAATTCCAGAAGATGTTGAAATAAAACTTAAAAAGATCGGAATTATTGAAACTGTACAAAATTCTCCATTTATGGCAATTCAAGCATTTACTAAATCTAACTATATTGATGTTGCTGAAGCATATTATAATAATAAACCGTTATCCTTCTATTCATATGATTCAATATATGATTGGAAAATAGATAAAGGAAATAAATTTATAATTACGGACGAAAGTGCG

**2. NRGp17 insertion sequence *(nluc:*CBM T4Δ*soc)* 2895 bp**

**ROH up hoc—nluc-linker (GSSG)—CBM—ROH down soc**

GATGTTGTTTCTTGGATGCGTGAACAAAAAGACTGTATTGATGATGAATTCCGCGAACTTCTGACTTCTCTTGGTGAAATGTCACGTGGTGAAAAAGAAGCTTCTGCTGTATGGAAAAAATGGAAAGCACGTTATATTGAAGCGCAAGAAAAACGCATTGATGAAATGTCCCCCGAAGACCAGCTCGAAATTAAATTTGAGCTTGTGGATATATTTCATTTCGTATTAAATATGTTTGTTGGCCTTGGAATGAATGCGGAAGAAATCTTTAAACTTTATTATCTGAAGAACAAACATAATTTTGAACGTCAAGATAATGGATATTAAACAAAAATTTTATAGAACATATATAGTAAAAGTTAGGACGCCGAAAGGCGTCTTTTGGTACGCTGGGAAACATGAATCATTTATTGTAAATCCATATAATGATAAATATCCGGGTTCAGGTAAAATACTATGGAACATATATCGTAAGTATGGATTTAATTATAAAATACGATGGTCAAAATGCCATGGTTCTAGAGAAAAATCATATGAAGTTGAACGTGAGCTAATATCTGCATTAAAACGTAAACACCCAGATACTTGCATTAATATTTCTCCTGGTGGTCAGGGTGGAGAAGGAAGAAAATGGACTGAGCAACAACGATTAGAACATAAACTTAGATTAAACAATCCTGAAACAAAAACTCGGATGAAGAATTCACAACGTATAGCCCAAAATAGGGCAGAAAGAAAAGCTCGGCAATCTGAAGTAATGAAAAAGTTTTATTCGAATGGCGGAAATAAAAAGATTTCAGAAGGAACTTCAAGGGCGCAAAGAAAAGCACCGCATTGGCATGAACCACTTAAAAGCGAAATACACGAGTTATGGGTTTCTTTAGGTAAACCAGCAACAGGCCCGGTTGTAAAGGCGCTTAAAGGAAAATATGATGTAACAAGTTCGGCTCTTAAGAATTTAATTTACTTATTCAGAAAAGAAGATGTATAAATAATCATGTAATTTAAATAAAGGAGAATTACATGGTATTCACTTTGGAGGATTTCGTAGGTGACTGGCGCCAGACAGCCGGTTACAATTTAGACCAAGTACTGGAACAGGGAGGTGTCAGTTCACTTTTTCAAAATTTGGGCGTTTCGGTTACACCCATCCAGCGCATTGTCCTTAGTGGAGAAAACGGGCTTAAAATCGACATCCATGTTATTATCCCCTATGAGGGGCTGTCGGGGGATCAGATGGGTCAGATTGAGAAAATCTTCAAAGTCGTCTACCCGGTTGATGACCATCACTTCAAGGTTATTCTTCATTACGGCACGCTGGTCATTGATGGCGTTACGCCTAATATGATTGACTACTTTGGCCGCCCCTATGAAGGGATCGCAGTGTTCGACGGCAAGAAGATCACTGTAACTGGAACATTGTGGAATGGCAATAAAATCATTGACGAACGTCTGATTAACCCGGACGGCTCTTTGCTGTTTCGTGTTACGATCAACGGGGTCACGGGCTGGCGTCTTTGTGAGCGCATTTTGGCTGGCTCGAGCGGCCCTACGTCAGGTCCGGCCGGTTGCCAAGTTTTATGGGGGGTCAACCAGTGGAACACAGGCTTTACGGCGAACGTTACTGTCAAGAACACAAGCTCCGCTCCTGTGGATGGTTGGACACTGACCTTTTCTTTCCCCTCAGGTCAGCAAGTGACACAGGCGTGGAGTTCTACGGTTACACAATCTGGTTCTGCTGTTACTGTCCGTAACGCGCCCTGGAATGGAAGCATCCCAGCGGGCGGGACCGCACAGTTTGGCTTCAATGGCTCTCATACAGGGACAAACGCAGCACCAACAGCATTTTCCTTGAATGGAACCCCTTGCACTGTCGGATAATAACTCAAGGACTCCTTCGGGAGTCCTTTTTTCATTTAAATGGTTTACTTTCCAAAATGAGTATGGTATAATAGAATTATCTTATAGAGGAGAGTACTATGTTAAATCGTTGGATTAAACCAAATGAAGATTTAGATATTATCATTTCACGACATGTAATGAAGAAATATGAACTACAACCATGGTCTACAGAAGTTGTTGTGCATTCATTTATGATGTACGCAGATGGTTCTGTCGAATTTAATGTAGAGATTCGATATGATTATGGTGAGAAGCAAGTCGAATTCAAAAGAGGCTTTTTGTAATGTTTATCTTTAATTGGTTTAAAAGTTTCTTTACGGATTTTTTCTCTACAACTCCTGGGGAAGGTGTAGTTCCTATTTCAAATGACTACCTTCCTTTAACTGTAGTTGAATATGTTTATATGGGAGATGGAACAGTAGAAGCAGTTACTATGACTTATGAAGAAGCCCAAGAATATTATAAAAATCCTTGGCGCTGGTCAACACCTACTACATCATCTAACACACAGAATACACAGTCTAGTTCTGATTCATATGATACTAATGTTCCTGTTCATGTATGGGCAGGTGATTCATGTGGAAGTTCTTGTGATTCTAGTTGTTCATCTACATCTTGTGATTGAGGAAAATTATGGAAGCAATTTTGTTTGAAATGTCTATCAATAGTATTAGTATGGCGTTTGTCAAGGATGTTCCAATTACTGTAGCAGTAATGATTGATAAAAGATATGACAATAATATGTATCTAGTAGAAGATTTTATTTCAATGCCAATTCCAGAAGATGTTGAAATAAAACTTAAAAAGATCGGAATTATTGAAACTGTACAAAATTCTCCATTTATGGCAATTCAAGCATTTACTAAATCTAACTATATTGATGTTGCTGAAGCATATTATAATAATAAACCGTTATCCTTCTATTCATATGATTCAATATATGATTGGAAAATAGATAAAGGAAATAAATTTATAATTACGGACGAAAGTGCG

**3. NRGp18 insertion sequence *(nluc:*CBM T4Δ*hoc)* 3859 bp**

**ROH up hoc—nluc-linker (GSSG)—CBM—ROH down hoc**

CCAGTTGCCATATCTGAAGCGAGGATTAAATCTATATCCATCGGCCTCAAACGAAAAGAAATCTCTTAATTCGTGGAACGTGCTCTCTTCACAATCGATGCGTACATGACTGAAGTCGTGAAAATGTACTTTAATATCCATAATTATGCCTTACTAAATTTGCCTTTAGAATCTCTTTTCATGAGACGACCTTTAATAAATCCGTCGGGAATAATACCGTCTGGTTGTATTAATTTATTTATTGCGCCATTATTGACCCAAAAAGTTCCTGTGGTAGTCGGTTTGACTTTACATCCTTTTCTGGACTTTCTATTAGGATGAACCATCCCTTTTACAAATCCTTCTGGAACAAGTTCTCCAGGTTTAATAAAAATATTTTTAGTTCCATTAGTATAACAAGTTTTACCTAATACCGTGCCCGGTGAGTTTTCAAATCTTTTAGCGGAAGATTCTTTCATCTTTGCTATAACATCTGTAGTCATAACAATTCCACCAATTCCACCAGGTTTCATATTATAATAATTTTTGCTTTTTATTAGTTCAGGAGTTATAATTTCTTCTTCATACATGTACGCTTCTTCGGAAGTTTTAAACTCTTTTAGTATTGTTCTAGAGAAATTGTTTTCACCATATTTCTTTATAGCCTGCTGAATTGCCTTACCGGAACCAAGGTAACCATCATTCAAGTCATCAGTAGAGTGCTTTCCTATATACTTTTTACCATTTATTAGATTTGTTGTTTCATATACAAAGTGGTACATACTATTTTCCGAGTAATAAATATATCTATATTTATACTGAGGAAATATTATGATAGATAAAGATTATATTGCAGAGCTGAAGGCTCTTGATGATAACAAAGAAGCTAAAGCTAAATTAGCTGAATATGCTGAACAGTTTGGTATAAAGGTCAAAAAGAATAAATCTTTTGATAATATCGTTGTTGATATTGAAGAAGCCCTCCAGAAGCTCGCTAGTGAACCTATGCCAGAGACTGATGGGTTATCTATTAAAGACTTAATTGATGCTGCTGATGCCGCAGAGGGATTAAAATATGACGATGAAGAAGTCAATCCAGAAGCAGCACTTCTGATTGATTCTCCGATTAAATCTGACATTAAAATTGAAGTAGTAGAAACGGATAAAATTCCTGAAAATACCGATGTTTTGATTGAAGATACTCCTTTTGTTGAAGAAAAATTCGAACAGGCTGTAGCTGAGATTATTGAATCTGAAAAGCCGTCTGTATTTACTCTTCCGGAAAACTTTAGTCCGAATCTTCAACTGATTGGAAAAAATCTAGGATTCTGTACTGTTCCTTGGTGGATTTATCAATGGATTGCTGAAACTCCTGATTGGAAATCTCACCCAACTAGTTTTGAACATGCGTCAGCACACCAAACTTTATTTAGCTTAATTTATTACATTAATCGCGATGGATCAGTTTTAATTCGTGAAACACGCAACTCTTCTTTCGTAACATTAAAATAAGGATAACTTATGGTATTCACTTTGGAGGATTTCGTAGGTGACTGGCGCCAGACAGCCGGTTACAATTTAGACCAAGTACTGGAACAGGGAGGTGTCAGTTCACTTTTTCAAAATTTGGGCGTTTCGGTTACACCCATCCAGCGCATTGTCCTTAGTGGAGAAAACGGGCTTAAAATCGACATCCATGTTATTATCCCCTATGAGGGGCTGTCGGGGGATCAGATGGGTCAGATTGAGAAAATCTTCAAAGTCGTCTACCCGGTTGATGACCATCACTTCAAGGTTATTCTTCATTACGGCACGCTGGTCATTGATGGCGTTACGCCTAATATGATTGACTACTTTGGCCGCCCCTATGAAGGGATCGCAGTGTTCGACGGCAAGAAGATCACTGTAACTGGAACATTGTGGAATGGCAATAAAATCATTGACGAACGTCTGATTAACCCGGACGGCTCTTTGCTGTTTCGTGTTACGATCAACGGGGTCACGGGCTGGCGTCTTTGTGAGCGCATTTTGGCTGGCTCGAGCGGCCCTACGTCAGGTCCGGCCGGTTGCCAAGTTTTATGGGGGGTCAACCAGTGGAACACAGGCTTTACGGCGAACGTTACTGTCAAGAACACAAGCTCCGCTCCTGTGGATGGTTGGACACTGACCTTTTCTTTCCCCTCAGGTCAGCAAGTGACACAGGCGTGGAGTTCTACGGTTACACAATCTGGTTCTGCTGTTACTGTCCGTAACGCGCCCTGGAATGGAAGCATCCCAGCGGGCGGGACCGCACAGTTTGGCTTCAATGGCTCTCATACAGGGACAAACGCAGCACCAACAGCATTTTCCTTGAATGGAACCCCTTGCACTGTCGGATAATCATAAGGGGCTTCGGCCCCTTTCTTCATTTTGAAAGCACACAAAACACAATCAGAAAATGATGTATATAATGGCACCAACTCGATAACATGAGATTGATTATGAGAACTGAGGTTGTGGTGTTTACTCTTCATGAGTCTGGAAAGTCATTCATTGAAATTGCTCGTGAATTAAACTTACAGGCAAAAGAAGTGGCTGTATTATGGGCTCGAGAAAAAGTTGTCTATAGAAAAAGACATATCAATAAAAAGGTGAAAAATGGAACAGTATGATCTTTATGAAAATGAATCTTTTGCTAATCAATTACGCGAAAAAGCATTAAAAAGTAAACAGTTTAAGCTAGAGTGTTTTATTAAAGATTTTTCAGAACTTGCTAATAAAGCAGCTGAACAAGGTAAAACACATTTTAATTATTATTGTATTGCTCGTGATAAATTGATTACAGAAGAAATTGGTGATTGGCTGAGAAAAGAAGGATTCAGCTTTAAAGTCAATAGTGATCAGCGTGATGGTGATTGGTTAGAAATTACATTTTGAGGATTAATTATGTTTAAAAAGTATAGCAGTCTTGAAAATCATTACAACTCTAAATTTATTGAAAAACTTTATAGCTTGGGATTGACTGGTGGGGAGTGGGTAGCTCGTGAAAAGATTCACGGCACAAATTTCTCATTGATTATTGAGCGTGATAAAGTGACTTGCGCTAAACGCACTGGACCGATTCTTCCTGCTGAAGATTTCTTTGGGTATGAAATTATTTTGAAGAATTATGCTGATTCCATTAAAGCTGTACAAGATATTATGGAAACCTCAGCGGTTGTATCTTATCAAGTCTTTGGCGAATTCGCTGGACCTGGCATTCAGAAGAATGTTGATTATTGTGATAAAGATTTTTATGTATTTGACATTATTGTTACTACAGAAAGCGGTGATGTGACTTATGTAGATGATTATATGATGGAATCATTCTGTAATACATTTAAATTTAAAATGGCTCCACTTTTAGGTCGCGGTAAATTTGAAGAGCTTATTAAATTGCCAAATGATTTAGATTCTGTCGTCCAAGATTATAATTTTACAGTAGACCATGCTGGATTAGTTGATGCAAATAAATGCGTTTGGAATGCCGAAGCAAAAGGCGAAGTATTTACTGCTGAAGGATATGTATTGAAACCTTGTTATCCTTCTTGGCTTCGTAATGGAAATCGTGTAGCGATTAAATGCAAGAACTCTAAATTTAGTGAAAAGAAAAAGTCTGATAAGCCTATTAAAGCTAAAGTTGAGCTATCAGAAGCTGATAACAAATTGGTGGGAATTTTAGCTTGTTACGTTACACTGAACCGCGTAAATAACGTTATTTCTAAAATTGGCGAAATTGGTCCAAAGGATTTTGGAAAGGTGATGGGGCTAACTGTTCAAGATATTTTGGAAGAAACTTCTCGTGAAGGTATTACTCTAACTCAAGCAGATAATCCTTCTTTGATTAAAAAGGAATTAGTTG

**4. NRGp20 insertion sequence *(*CBM:Hoc T4*)* 4472 bp**

**ROH up hoc—CBM-linker (PGG)—Hoc-ROH down hoc**

CAGTTGCCATATCTGAAGCGAGGATTAAATCTATATCCATCGGCCTCAAACGAAAAGAAATCTCTTAATTCGTGGAACGTGCTCTCTTCACAATCGATGCGTACATGACTGAAGTCGTGAAAATGTACTTTAATATCCATAATTATGCCTTACTAAATTTGCCTTTAGAATCTCTTTTCATGAGACGACCTTTAATAAATCCGTCGGGAATAATACCGTCTGGTTGTATTAATTTATTTATTGCGCCATTATTGACCCAAAAAGTTCCTGTGGTAGTCGGTTTGACTTTACATCCTTTTCTGGACTTTCTATTAGGATGAACCATCCCTTTTACAAATCCTTCTGGAACAAGTTCTCCAGGTTTAATAAAAATATTTTTAGTTCCATTAGTATAACAAGTTTTACCTAATACCGTGCCCGGTGAGTTTTCAAATCTTTTAGCGGAAGATTCTTTCATCTTTGCTATAACATCTGTAGTCATAACAATTCCACCAATTCCACCAGGTTTCATATTATAATAATTTTTGCTTTTTATTAGTTCAGGAGTTATAATTTCTTCTTCATACATGTACGCTTCTTCGGAAGTTTTAAACTCTTTTAGTATTGTTCTAGAGAAATTGTTTTCACCATATTTCTTTATAGCCTGCTGAATTGCCTTACCGGAACCAAGGTAACCATCATTCAAGTCATCAGTAGAGTGCTTTCCTATATACTTTTTACCATTTATTAGATTTGTTGTTTCATATACAAAGTGGTACATACTATTTTCCGAGTAATAAATATATCTATATTTATACTGAGGAAATATTATGATAGATAAAGATTATATTGCAGAGCTGAAGGCTCTTGATGATAACAAAGAAGCTAAAGCTAAATTAGCTGAATATGCTGAACAGTTTGGTATAAAGGTCAAAAAGAATAAATCTTTTGATAATATCGTTGTTGATATTGAAGAAGCCCTCCAGAAGCTCGCTAGTGAACCTATGCCAGAGACTGATGGGTTATCTATTAAAGACTTAATTGATGCTGCTGATGCCGCAGAGGGATTAAAATATGACGATGAAGAAGTCAATCCAGAAGCAGCACTTCTGATTGATTCTCCGATTAAATCTGACATTAAAATTGAAGTAGTAGAAACGGATAAAATTCCTGAAAATACCGATGTTTTGATTGAAGATACTCCTTTTGTTGAAGAAAAATTCGAACAGGCTGTAGCTGAGATTATTGAATCTGAAAAGCCGTCTGTATTTACTCTTCCGGAAAACTTTAGTCCGAATCTTCAACTGATTGGAAAAAATCTAGGATTCTGTACTGTTCCTTGGTGGATTTATCAATGGATTGCTGAAACTCCTGATTGGAAATCTCACCCAACTAGTTTTGAACATGCGTCAGCACACCAAACTTTATTTAGCTTAATTTATTACATTAATCGCGATGGATCAGTTTTAATTCGTGAAACACGCAACTCTTCTTTCGTAACATTAAAATAAGGATAACTTATGCCTACGTCAGGTCCGGCCGGTTGCCAAGTTTTATGGGGGGTCAACCAGTGGAACACAGGCTTTACGGCGAACGTTACTGTCAAGAACACAAGCTCCGCTCCTGTGGATGGTTGGACACTGACCTTTTCTTTCCCCTCAGGTCAGCAAGTGACACAGGCGTGGAGTTCTACGGTTACACAATCTGGTTCTGCTGTTACTGTCCGTAACGCGCCCTGGAATGGAAGCATCCCAGCGGGCGGGACCGCACAGTTTGGCTTCAATGGCTCTCATACAGGGACAAACGCAGCACCAACAGCATTTTCCTTGAATGGAACCCCTTGCACTGTCGGACCAGGCGGCATGACTTTTACAGTTGATATAACTCCTAAAACACCTACTGGTGTAATAGACGAGACTAAGCAGTTTACTGCTACACCCAGTGGTCAAACTGGAGGCGGAACTATTACATATGCTTGGAGCGTAGATAATGTTCCACAAGATGGAGCTGAAGCAACTTTTAGTTATGTACTAAAAGGACCTGCCGGTCAAAAGACTATTAAAGTAGTTGCAACAAATACACTTTCTGAAGGAGGCCCGGAAACGGCTGAAGCGACAACAACTATCACAGTTAAAAATAAGACACAGACGACTACCTTAGCCGTAACTCCTGCTAGTCCTGCGGCTGGAGTGATTGGAACCCCAGTTCAATTTACTGCTGCCTTAGCTTCTCAACCTGATGGAGCATCTGCTACGTATCAGTGGTATGTAGATGATTCACAAGTTGGTGGAGAAACTAACTCTACATTTAGCTATACTCCAACTACAAGTGGAGTAAAAAGAATTAAATGCGTAGCCCAAGTAACCGCGACAGATTATGATGCACTAAGCGTTACTTCTAATGAAGTATCATTAACGGTTAATAAGAAGACAATGAATCCACAGGTTACATTGACTCCTCCTTCTATTAATGTTCAGCAAGATGCTTCGGCTACATTTACGGCTAATGTTACGGGTGCTCCAGAAGAAGCACAAATTACTTACTCATGGAAGAAAGATTCTTCTCCTGTAGAAGGGTCAACTAACGTATATACTGTCGATACCTCATCTGTTGGAAGTCAAACTATTGAAGTTACTGCAACTGTTACTGCTGCAGATTATAACCCTGTAACCGTTACCAAAACTGGTAATGTAACAGTCACGGCTAAAGTTGCTCCAGAACCAGAAGGTGAATTACCTTATGTTCATCCTCTTCCACACCGTAGCTCAGCTTACATCTGGTGCGGTTGGTGGGTTATGGATGAAATCCAAAAAATGACCGAAGAAGGTAAAGATTGGAAAACTGACGACCCAGATAGTAAATATTACCTGCATCGTTACACTCTCCAGAAGATGATGAAAGACTATCCAGAAGTTGATGTTCAAGAATCGCGTAATGGATACATCATTCATAAAACTGCTTTAGAAACTGGTATCATCTATACCTATCCATAATCATAAGGGGCTTCGGCCCCTTTCTTCATTTTGAAAGCACACAAAACACAATCAGAAAATGATGTATATAATGGCACCAACTCGATAACATGAGATTGATTATGAGAACTGAGGTTGTGGTGTTTACTCTTCATGAGTCTGGAAAGTCATTCATTGAAATTGCTCGTGAATTAAACTTACAGGCAAAAGAAGTGGCTGTATTATGGGCTCGAGAAAAAGTTGTCTATAGAAAAAGACATATCAATAAAAAGGTGAAAAATGGAACAGTATGATCTTTATGAAAATGAATCTTTTGCTAATCAATTACGCGAAAAAGCATTAAAAAGTAAACAGTTTAAGCTAGAGTGTTTTATTAAAGATTTTTCAGAACTTGCTAATAAAGCAGCTGAACAAGGTAAAACACATTTTAATTATTATTGTATTGCTCGTGATAAATTGATTACAGAAGAAATTGGTGATTGGCTGAGAAAAGAAGGATTCAGCTTTAAAGTCAATAGTGATCAGCGTGATGGTGATTGGTTAGAAATTACATTTTGAGGATTAATTATGTTTAAAAAGTATAGCAGTCTTGAAAATCATTACAACTCTAAATTTATTGAAAAACTTTATAGCTTGGGATTGACTGGTGGGGAGTGGGTAGCTCGTGAAAAGATTCACGGCACAAATTTCTCATTGATTATTGAGCGTGATAAAGTGACTTGCGCTAAACGCACTGGACCGATTCTTCCTGCTGAAGATTTCTTTGGGTATGAAATTATTTTGAAGAATTATGCTGATTCCATTAAAGCTGTACAAGATATTATGGAAACCTCAGCGGTTGTATCTTATCAAGTCTTTGGCGAATTCGCTGGACCTGGCATTCAGAAGAATGTTGATTATTGTGATAAAGATTTTTATGTATTTGACATTATTGTTACTACAGAAAGCGGTGATGTGACTTATGTAGATGATTATATGATGGAATCATTCTGTAATACATTTAAATTTAAAATGGCTCCACTTTTAGGTCGCGGTAAATTTGAAGAGCTTATTAAATTGCCAAATGATTTAGATTCTGTCGTCCAAGATTATAATTTTACAGTAGACCATGCTGGATTAGTTGATGCAAATAAATGCGTTTGGAATGCCGAAGCAAAAGGCGAAGTATTTACTGCTGAAGGATATGTATTGAAACCTTGTTATCCTTCTTGGCTTCGTAATGGAAATCGTGTAGCGATTAAATGCAAGAACTCTAAATTTAGTGAAAAGAAAAAGTCTGATAAGCCTATTAAAGCTAAAGTTGAGCTATCAGAAGCTGATAACAAATTGGTGGGAATTTTAGCTTGTTACGTTACACTGAACCGCGTAAATAACGTTATTTCTAAAATTGGCGAAATTGGTCCAAAGGATTTTGGAAAGGTGATGGGGCTAACTGTTCAAGATATTTTGGAAGAAACTTCTCGTGAAGGTATTACTCTAACTCAAGCAGATAATCCTTCTTTGATTAAAAAGGAATTAGTT

Table S3. ImageJ Analysis of Recombination Frequency

ImageJ Analysis of Positive Recombination Counts for *soc*/HR

| Slice (plate) | Count (Max 36/slice) | Total Area | Average Particle Size | % Area |
| --- | --- | --- | --- | --- |
| soc-HR-1 | 0 | 0 | 0 | 0 |
| soc-HR-2 | 1 | 7120 | 7120 | 0.29 |
| soc-HR-3 | 1 | 421 | 421 | 0.017 |
| soc-HR-4 | 0 | 0 | 0 | 0 |
| soc-HR-5 | 0 | 0 | 0 | 0 |
| soc-HR-6 | 0 | 0 | 0 | 0 |
| soc-HR-7 | 1 | 520 | 520 | 0.021 |
| soc-HR-8 | 0 | 0 | 0 | 0 |
| soc-HR-9 | 1 | 949 | 949 | 0.039 |
| soc-HR-10 | 1 | 415 | 415 | 0.017 |
| soc-HR-11 | 0 | 0 | 0 | 0 |
| soc-HR-12 | 3 | 5789 | 1929.667 | 0.236 |
| soc-HR-13 | 1 | 1980 | 1980 | 0.081 |
| soc-HR-14 | 0 | 0 | 0 | 0 |
| Total | **9/504** |  |  |  |

ImageJ Analysis of Positive Recombination Counts for *soc/*CRISPR (5 log EOP gRNA)/HR

|  |
| --- |

| Slice (plate) | Count (Max 36/slice) | Total Area | Average Particle Size | % Area |
| --- | --- | --- | --- | --- |
| soc-CRISPR/HR-1 | 36 | 154674 | 4296.5 | 6.294 |
| soc-CRISPR/HR-2 | 36 | 178615 | 4961.528 | 7.268 |
| soc-CRISPR/HR-3 | 36 | 169290 | 4702.5 | 6.888 |
| soc-CRISPR/HR-4 | 35 | 160874 | 4596.4 | 6.546 |
| soc-CRISPR/HR-5 | 36 | 159446 | 4429.056 | 6.488 |
| soc-CRISPR/HR-6 | 35 | 161097 | 4602.771 | 6.555 |
| soc-CRISPR/HR-7 | 36 | 137910 | 3830.833 | 5.612 |
| soc-CRISPR/HR-8 | 36 | 182661 | 5073.917 | 7.432 |
| soc-CRISPR/HR-9 | 36 | 174572 | 4849.222 | 7.103 |
| soc-CRISPR/HR-10 | 36 | 164310 | 4564.167 | 6.686 |
| soc-CRISPR/HR-11 | 36 | 220094 | 6113.722 | 8.956 |
| soc-CRISPR/HR-12 | 36 | 226490 | 6291.389 | 9.216 |
| soc-CRISPR/HR-13 | 36 | 167747 | 4659.639 | 6.826 |
| soc-CRISPR/HR-14 | 36 | 177850 | 4940.278 | 7.237 |
| Total | **502/503** |  |  |  |

ImageJ Analysis of Positive Recombination Counts for *hoc*/HR

| Slice (plate) | Count (Max 36/slice) | Total Area | Average Particle Size | % Area |
| --- | --- | --- | --- | --- |
| hoc-HR-1 | 2 | 6569 | 3284.5 | 0.267 |
| hoc-HR-2 | 7 | 12468 | 1781.143 | 0.507 |
| hoc-HR-3 | 7 | 18098 | 2585.429 | 0.736 |
| hoc-HR-4 | 3 | 4085 | 1361.667 | 0.166 |
| hoc-HR-5 | 0 | 0 | 0 | 0 |
| hoc-HR-6 | 3 | 2166 | 722 | 0.088 |
| hoc-HR-7 | 5 | 11600 | 2320 | 0.472 |
| hoc-HR-8 | 12 | 23765 | 1980.417 | 0.967 |
| hoc-HR-9 | 8 | 18928 | 2366 | 0.77 |
| hoc-HR-10 | 5 | 11319 | 2263.8 | 0.461 |
| hoc-HR-11 | 6 | 4995 | 832.5 | 0.203 |
| hoc-HR-12 | 7 | 19813 | 2830.429 | 0.806 |
| hoc-HR-13 | 6 | 14114 | 2352.333 | 0.574 |
| hoc-HR-14 | 3 | 2895 | 965 | 0.118 |
| Total | **74/502** |  |  |  |

ImageJ Analysis of Positive Recombination Counts for *hoc/*CRISPR (2 log EOP gRNA)/HR

| Slice (plate) | Count (Max 36/slice) | Total Area | Average Particle Size | % Area |
| --- | --- | --- | --- | --- |
| hoc-CRISPR/HR-1 | 36 | 116663 | 3240.639 | 4.747 |
| hoc-CRISPR/HR-2 | 34 | 134407 | 3953.147 | 5.469 |
| hoc-CRISPR/HR-3 | 36 | 132567 | 3682.417 | 5.394 |
| hoc-CRISPR/HR-4 | 36 | 109684 | 3046.778 | 4.463 |
| hoc-CRISPR/HR-5 | 35 | 103164 | 2947.543 | 4.198 |
| hoc-CRISPR/HR-6 | 36 | 148596 | 4016.108 | 6.046 |
| hoc-CRISPR/HR-7 | 32 | 225064 | 7033.25 | 9.158 |
| hoc-CRISPR/HR-8 | 36 | 222070 | 6168.611 | 9.036 |
| hoc-CRISPR/HR-9 | 36 | 214191 | 5949.75 | 8.715 |
| hoc-CRISPR/HR-10 | 36 | 177154 | 4920.944 | 7.208 |
| hoc-CRISPR/HR-11 | 36 | 217783 | 6049.528 | 8.862 |
| hoc-CRISPR/HR-12 | 36 | 218548 | 6070.778 | 8.893 |
| hoc-CRISPR/HR-13 | 36 | 128334 | 3564.833 | 5.222 |
| hoc-CRISPR/HR-14 | 33 | 261704 | 7930.424 | 10.649 |
| total | **494/496** |  |  |  |
